# Mind the gap: Performance metric evaluation in brain‐age prediction

**DOI:** 10.1002/hbm.25837

**Published:** 2022-03-21

**Authors:** Ann‐Marie G. de Lange, Melis Anatürk, Jaroslav Rokicki, Laura K. M. Han, Katja Franke, Dag Alnæs, Klaus P. Ebmeier, Bogdan Draganski, Tobias Kaufmann, Lars T. Westlye, Tim Hahn, James H. Cole

**Affiliations:** ^1^ LREN, Centre for Research in Neurosciences, Department of Clinical Neurosciences Lausanne University Hospital (CHUV) and University of Lausanne Lausanne; ^2^ Department of Psychology University of Oslo Oslo; ^3^ Department of Psychiatry University of Oxford Oxford; ^4^ Centre for Medical Image Computing, Department of Computer Science University College London London UK; ^5^ NORMENT, Institute of Clinical Medicine University of Oslo, & Division of Mental Health and Addiction, Oslo University Hospital Oslo Norway; ^6^ Centre of Research and Education in Forensic Psychiatry Oslo University Hospital Oslo Norway; ^7^ Department of Psychiatry Amsterdam University Medical Centers, Vrije Universiteit and GGZ inGeest, Amsterdam Neuroscience Amsterdam The Netherlands; ^8^ Structural Brain Mapping Group, Department of Neurology Jena University Hospital Jena Germany; ^9^ Department of Neurology Max Planck Institute for Human Cognitive and Brain Sciences Leipzig Germany; ^10^ Tübingen Center for Mental Health, Department of Psychiatry and Psychotherapy University of Tübingen Tübingen Germany; ^11^ KG Jebsen Centre for Neurodevelopmental Disorders University of Oslo Oslo Norway; ^12^ Institute of Translational Psychiatry University of Münster Münster Germany; ^13^ Dementia Research Centre, Queen Square Institute of Neurology University College London London UK

**Keywords:** brain‐age prediction, machine learning, neuroimaging, statistics

## Abstract

Estimating age based on neuroimaging‐derived data has become a popular approach to developing markers for brain integrity and health. While a variety of machine‐learning algorithms can provide accurate predictions of age based on brain characteristics, there is significant variation in model accuracy reported across studies. We predicted age in two population‐based datasets, and assessed the effects of age range, sample size and age‐bias correction on the model performance metrics Pearson's correlation coefficient (*r*), the coefficient of determination (*R* ^2^), Root Mean Squared Error (RMSE) and Mean Absolute Error (MAE). The results showed that these metrics vary considerably depending on cohort age range; *r* and *R* ^2^ values are lower when measured in samples with a narrower age range. RMSE and MAE are also lower in samples with a narrower age range due to smaller errors/brain age delta values when predictions are closer to the mean age of the group. Across subsets with different age ranges, performance metrics improve with increasing sample size. Performance metrics further vary depending on prediction variance as well as mean age difference between training and test sets, and age‐bias corrected metrics indicate high accuracy—also for models showing poor initial performance. In conclusion, performance metrics used for evaluating age prediction models depend on cohort and study‐specific data characteristics, and cannot be directly compared across different studies. Since age‐bias corrected metrics generally indicate high accuracy, even for poorly performing models, inspection of uncorrected model results provides important information about underlying model attributes such as prediction variance.

## Graphical abstract

While a variety of machine‐learning algorithms can provide accurate predictions of age based on brain characteristics, there is significant variation in model accuracy reported across studies. We predicted age based on neuroimaging data in two population‐based datasets, and assessed the effects of age range, sample size, and age‐bias correction on the model performance metrics *r*, *R* ^2^, Root Mean Squared Error, and Mean Absolute Error. The results showed that these metrics depend on cohort and study‐specific data characteristics including age range and sample size, and cannot be directly compared across different studies. Age‐bias corrected metrics indicate high accuracy, even for poorly performing models, and inspection of uncorrected model results thus provides important information about underlying model attributes such as prediction variance.

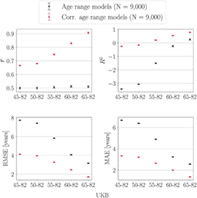

## 1 INTRODUCTION

Brain‐predicted age is increasingly used as a marker for structural brain integrity and health across normative and clinical populations (Bittner et al., 2021; Cole, 2020; Cole et al., 2018, 2020; de Lange et al., 2019, 2020; Franke, Gaser, & Alzheimer's Disease Neuroimaging Initiative, 2012; Franke, Gaser, Manor, & Novak, 2013; Franke, Ristow, & Gaser, 2014; Gaser et al., 2013; Høgestøl et al., 2019; Kaufmann et al., 2019; Pardoe et al., 2017; Richard et al., 2019; Rokicki et al., 2021; Schnack et al., 2016; Smith et al., 2020). Since brain structure is known to vary with age across the lifespan, machine learning (ML) regression models can be used to predict chronological age based on neuroimaging data (Cole et al., 2017; Cole & Franke, 2017; Cole, Franke, & Cherbuin, 2019; Franke et al., 2010; Franke & Gaser, 2019). Training a regression model on a wide range of magnetic resonance imaging (MRI) scans allows it to build a normative trajectory of brain differences across age, and condense a rich variety of brain characteristics into a single quantity per individual. Prediction models can then be applied to unseen data, providing an estimate of brain‐predicted age for each individual in the dataset. The difference between an individual's brain‐predicted and chronological age (*brain age delta*) provides a proxy for deviations from expected age trajectories, and has been associated with clinical risk factors (Beck et al., 2022; Cole, 2020; de Lange, Anatürk, et al., 2020) as well as neurological and neuropsychiatric conditions (Cole et al., 2020; Cole, Marioni, Harris, & Deary, 2019; Franke & Gaser, 2019; Hajek et al., 2019; Han et al., 2020; Kaufmann et al., 2019; Kolenic et al., 2018; Rokicki et al., 2021; Tønnesen et al., 2020; Van Gestel et al., 2019). Brain age delta estimates have also been linked to biomedical variables and lifestyle factors in healthy population cohorts (Anatürk et al., 2021; Cole, 2020; Cole, Franke, & Cherbuin, 2019; de Lange et al., 2019; Dunås, Wåhlin, Nyberg, & Boraxbekk, 2021; Franke et al., 2020; Smith et al., 2020), and the overall evidence supports the use of brain‐predicted age as a surrogate marker for brain integrity and health (Cole et al., 2017).

A number of recent studies show that ML algorithms can predict age based on MRI data with high accuracy, for example, (Couvy‐Duchesne et al., 2020; Gong, Beckmann, Vedaldi, Smith, & Peng, 2021; Han et al., 2020; Kaufmann et al., 2019, Leonardsen et al., 2021). However, in addition to differences in feature sets included (Cole, 2020; de Lange, Anatürk, et al., 2020; Jollans et al., 2019), training and test set characteristics such as size and age range (de Lange, Anatürk, et al., 2020; Jollans et al., 2019) can lead to considerable variation in model performance metrics across studies. This is due to general statistical features of regression models, and is not specific to brain‐age prediction. Prediction accuracy is commonly evaluated using the correlation coefficient for brain‐predicted versus chronological age (*r*), or the coefficient of determination (*R* ^2^), in addition to Root Mean Squared Error (RMSE) and Mean Absolute Error (MAE). While these metrics are useful for comparing different algorithms applied to the same dataset, the comparison of model performance across studies is less straightforward. For example, the correlation coefficient is reduced when measured in restricted ranges of a variable (Bland & Altman, 2011; Bryant & Gokhale, 1972), while the model error metrics RMSE and MAE depend on the distribution of the predicted variable, and will thus vary between studies with different cohort age ranges.

In age‐prediction studies, statistical corrections of overestimated predictions in younger subjects and underestimated predictions in older subjects can also have a large effect on model performance metrics. This phenomenon, which is commonly referred to as age‐bias (Beheshti, Nugent, Potvin, & Duchesne, 2019; de Lange & Cole, 2020; Le et al., 2018; Liang, Zhang, & Niu, 2019; Smith, Vidaurre, Alfaro‐Almagro, Nichols, & Miller, 2019), occurs due to general statistical features of a regression analysis (see Section 2.6). Age‐bias correction ensures that any group comparisons or associations with other variables of interest are not influenced by the age‐dependence of the predictions. However, model performance metrics calculated post correction may not always provide a relevant or valid representation of the initial model performance. This is important since the validity of brain‐predicted age estimates depends on aspects such as sufficient variance in predictions, which is contingent on how well the initial model performs.

With an increasing number of studies using brain age prediction based on ML regression models, there is a pressing need to establish a general understanding of model performance metrics, and how and why they may vary across studies. In this work, we address general statistical aspects of regression models in a brain‐age specific context, and demonstrate the effects of age range, sample size and age‐bias correction on metrics that are commonly used to evaluate model accuracy; *r*, *R* ^2^, RMSE and MAE.

## 2 MATERIALS AND METHODS

### 2.1 Datasets and data availability

The data were derived from UK Biobank (UKB) and the Cambridge Centre for Ageing and Neuroscience dataset (Cam‐CAN). Sample demographics are provided in Table 1. The two datasets were chosen due to large sample size (UKB) and wide age range (Cam‐CAN). The data are available through established access procedures for UKB (https://www.ukbiobank.ac.uk/researchers) and Cam‐CAN (https://www.cam-can.org/index.php?content=dataset). The code used for running the age prediction models is available at https://github.com/amdelange/brainage.

**TABLE 1:** Sample demographics

	UKB	Cam‐CAN
***N***	41,285	622
**Age**		
Mean ± *SD*	64.15 ± 7.54	54.17 ± 18.40
Range (years)	45–82	18–87
**Sex**		
% male	47.36	50.64
% female	52.64	49.35
**Scanner site**		
% 1	25.19	100
% 2	61.48	0
% 3	13.33	0

*Note*: For UKB, scanner site 1 represents Newcastle, site 2 and 3 represents Cheadle and Reading, respectively (all UKB sites use 3 T Siemens Skyra scanners with 32‐channel head coils). Mean age ± *SD* for each of the UKB sites: 1 = 64.90 ± 7.41; 2 = 63.47 ± 7.50; 3 = 65.81 ± 7.55. Sex distribution (M/F): 1 = 45.73/54.27%, 2 = 48.23/31.83%, 3 = 46.47/53.53%. For Cam‐CAN, site 1 represents Cambridge (3 T Siemens TIM Trio with a 32‐channel head coil).

### 2.2 MRI data acquisition and processing

A detailed overview of the UKB data acquisition and protocols is provided in (Alfaro‐Almagro et al., 2018; Miller et al., 2016), and the processing pipeline is available in (Kaufmann et al., 2019). For Cam‐CAN, study protocols are available in Shafto et al. (2014) and Taylor et al. (2017). For each of the datasets, global and regional measures of cortical volume, area and thickness in addition to subcortical volume were extracted based on the Desikan–Killiany atlas (Desikan et al., 2006) and automatic subcortical segmentation in FreeSurfer (version 5.3; Fischl et al., 2002). This set of features include MRI measures that are generally found to change with age (Storsve et al., 2014; Walhovd et al., 2005), and have been used in previous global and regional age prediction models (de Lange et al., 2020; Kaufmann et al., 2019; Smith et al., 2020; for details, see https://surfer.nmr.mgh.harvard.edu/fswiki/CorticalParcellation, www.frontiersin.org/articles/10.3389/fnins.2012.00171/full#h12 and https://freesurfer.net/fswiki/SubcorticalSegmentation). For UKB, the MRI data were residualised with respect to scanning site (Alfaro‐Almagro et al., 2021; Solanes et al., 2021) using linear models. To remove poor‐quality data likely due to subject motion, UKB participants with Euler numbers (Rosen et al., 2018) of ≥3 *SD*s from the mean were identified and excluded (*N* = 778; de Lange, Barth, et al., 2020). For Cam‐CAN, 28 participants were excluded based on manual inspection of images as described in Beck et al. (2022) and Richard et al. (2018). In total, data from 41,285 and 622 participants were included for UKB and Cam‐CAN, respectively.

### 2.3 Brain‐age prediction

To estimate global brain age, we used the *XGBoost* regression algorithm (XGB; https://github.com/dmlc/xgboost), which is based on gradient tree boosting. XGB has demonstrated high performance in previous machine learning competitions (Chen & Guestrin, 2016), and has been used in a number of recent brain age studies (Anatürk et al., 2021; Beck et al., 2021; de Lange et al., 2019; de Lange, Anatürk, et al., 2020; Voldsbekk et al., 2021; Richard et al., 2020). Learning objective was set to regression with squared loss. To test whether choice of algorithm influenced the results, we repeated the UKB analyses in Sections 2.5 and 2.6 using Linear Support Vector Regression (SVR; https://scikit-learn.org/stable/modules/generated/sklearn.svm.LinearSVR.html) with loss = *epsilon insensitive*. Hyper parameters for both algorithms were tuned in a held‐out UKB subset (*N* = 4,129) using nested cross‐validation with three inner folds for randomised search, and five outer folds for model validation. Subsequent models were run for (i) the rest of the UKB sample (*N* = 37,156) and the full Cam‐CAN sample (*N* = 622), (ii) UKB subsets with different age range and sample sizes (see Section 2.5) and (iii) UKB and Cam‐CAN samples where fractions of the data were randomly shuffled (see Section 2.6). For each iteration, the MRI input features were scaled using the robust scaler (Baecker et al., 2021) from the scikit‐learn library (Pedregosa et al., 2011), which removes the median and scales the data according to the quantile range.

### 2.4 Model performance metrics

Model performance metrics included the correlation between brain‐predicted and chronological age (Pearson's *r*), *R* ^2^, RMSE and MAE. An overview is provided in Table 2. For all models, uncertainties on the metrics were calculated using 200 bootstraps of each sample.

**TABLE 2:** Overview of the model performance metrics and how they are usually interpreted in the context of model accuracy (italic font)

Metric	Description	Equation
*r*	The correlation coefficient (here, Pearson's *r*) between predicted and chronological age. *Higher values indicate better fit*.	$r=\frac{\sum \left(y_{i}-\bar{y}\right)\left({\hat{y}}_{i}-\bar{\hat{y}}\right)}{\sqrt{\sum {\left(y_{i}-\bar{y}\right)}^{2}\sum {\left({\hat{y}}_{i}-\bar{\hat{y}}\right)}^{2}}}$
*R* ^2^	The proportion of the variance in the dependent variable that can be explained by the independent variables (not equivalent to *r* squared). *Higher values indicate better fit*.	$R^{2}=1-\frac{\sum_{i=1}^{N}{\left({\hat{y}}_{i}-y_{i}\right)}^{2}}{\sum_{i=1}^{N}{\left(\bar{y}-y_{i}\right)}^{2}}$
RMSE	The square root of the average of squared errors, which provides an overall measure of the prediction error across the group. *Lower values indicate better fit*.	$\text{RMSE}=\sqrt{\frac{1}{N}\sum_{i=1}^{N}{\left({\hat{y}}_{i}-y_{i}\right)}^{2}}$
MAE	The average of the absolute value of each residual; similar to RMSE as an overall measure of the prediction error across the group. *Lower values indicate better fit*.	$\mathrm{MAE}=\frac{1}{N}\sum_{i=1}^{N}\left|{\hat{y}}_{i}-y_{i}\right|$

*Note*: Here, *y* are the true age values for each subject, $\hat{y}$ are their predicted age values, $\bar{y}$ is the mean true age of the sample and $\bar{\hat{y}}$ is the mean predicted age of the sample.

Abbreviations: MAE, mean absolute error; RMSE, root mean squared error.

### 2.5 Effects of age range and sample size

To assess the effects of age range and sample size, we ran a series of experimental tests as described in the sections below. Due to the large sample size, UKB data were used to systematically assess effects of age range and sample size using subsets as described in Sections 2.5.2 and 2.5.3.

#### 2.5.1 Dataset comparison: full age range and sample size in each cohort

To compare the performance metrics for general models based on UKB versus Can‐CAN data, we ran models including the full age range (45–82 years for UKB; 18–87 years for Cam‐CAN) and sample size (*N* = 37,156 for UKB; 622 for Cam‐CAN) within each dataset. To maximise the statistics on which the performance metrics were based, 10‐fold cross‐validation was used. This procedure splits the sample into 10 folds of random subsets, where 9 of the 10 folds are used to train the model and predictions for the remaining fold are then made. Age distributions for 10 random folds in each dataset are shown in Figure S1. This process is repeated 10 times, with a different fold held out of the training each time, in order to generate predictions for all subjects.

#### 2.5.2 Test sets with varying age ranges; training set held constant

To assess the performance metrics in test sets with different age ranges, we trained a model on a subset including the full age range, and applied it to unseen test sets with different age ranges. For this experiment, a random 50/50 split was first applied to the full dataset, where one half of the data served as the training set, and the other half was used to create test sets with different age ranges. In this setting, age range varies only for the test sets. Sample size was held constant across training and test sets with *N* representing the maximum number of participants available with the narrowest age range.

#### 2.5.3 Training sets with varying age ranges; test set held constant

To assess the performance metrics when age range was varied only for the training sets, we trained models based on subsets with different age ranges, and applied them to the same test set. For this experiment, a random 50/50 split was first applied to the full dataset, where one half of the data was used to create training sets with different age ranges. The other half was used to select the test set, where an age range cut was applied to retain only the subjects within the narrowest age range. Sample size was held constant across training and test sets with *N* representing the maximum number of participants available with the narrowest age range.

#### 2.5.4 Training and test sets with equal age ranges

To assess the performance metrics when age range was equal for training and test sets, we ran models using 10‐fold cross‐validation within a series of subsets with different age ranges. To test the effects of age range in addition to sample size, we also ran the cross‐validation models using fractions of 2.5, 5, 10, 25, 50, 75 and 100% of the maximum number of participants available within the narrowest age range.

### 2.6 Age‐bias correction

**FIGURE 1:**
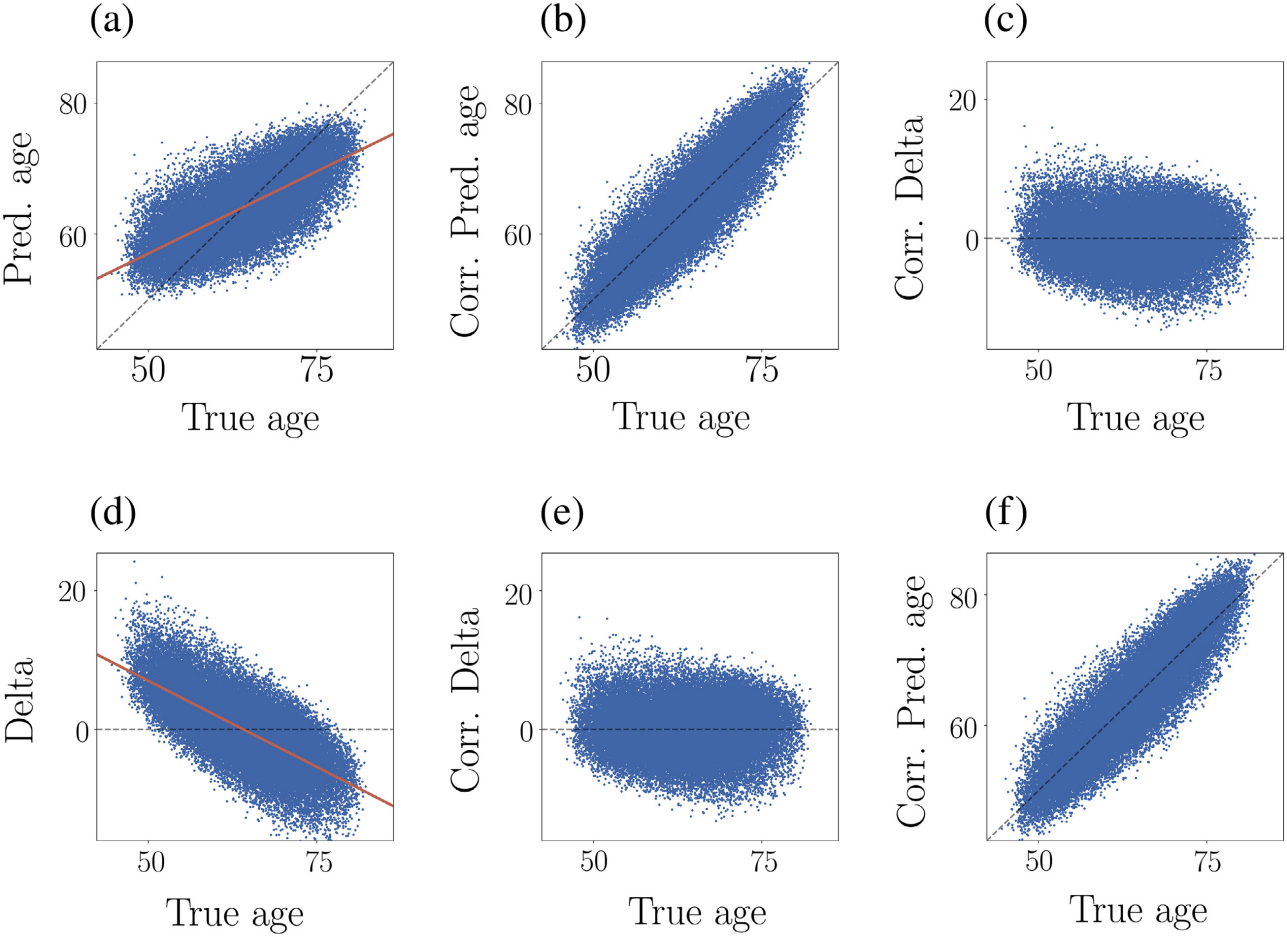
Example of age‐bias correction applied to (i) predicted age (top row) and (ii) brain age delta (bottom row). (a) The uncorrected association between predicted and true age. The orange line shows the linear fit applied to model the age bias. (b) The relationship between predicted and true age after using the coefficients from the fit (orange line in plot a) to correct predicted age. (c) Corrected delta calculated as *corrected predicted age* − *true age*, which shows no age dependence. (d) The uncorrected relationship between brain age delta and true age, with the orange line showing the linear fit applied to model the age bias. The negative slope is due to an anti‐correlation between true age on the *x*‐axis and negative true age on the *y*‐axis, which occurs since negative true age is part of delta (*predicted age − true age*). (e) Corrected delta calculated based on the correction in plot d, which shows no age dependence. (f) Corrected predicted age calculated using *corrected delta + true age*. Hence, corrected delta obtained via a correction of the predicted age values gives equivalent results to correcting the delta values themselves for age (de Lange & Cole, 2020), since the delta value contains the prediction minus true age. The correlation (*r*) between the corrected delta values in plots c and e = 1.00

Brain‐predicted age is often overestimated in younger subjects and underestimated in older subjects due to general statistical features of the regression analysis (Liang et al., 2019). This phenomenon can be explained by the limiting case where a model is unable to predict age based on the input features. In this scenario, all subjects will be predicted to have the median age (equivalent to the mean age if the data are symmetrically distributed), because such an estimate minimises the residuals; this is the aim of regression/ordinary least squares fitting. Assigning the median age as the prediction for all subjects will overestimate young subjects and underestimate older subjects (see Figure 1, and Figure 9 in Section 3). With increasing prediction accuracy, the degree to which the model predicts median age is reduced, since the predictions move closer to true age. Hence, age‐bias is less pronounced in models with high prediction accuracy, but will always be present to some extent since the relationship between brain characteristics and age is not perfect (as in *x* = *y*). To account for the method‐inherent age‐bias, a statistical correction can be applied to the age predictions or brain age delta estimates (Beheshti et al., 2019; Cole, 2020; de Lange et al., 2019; Gong et al., 2021; Le et al., 2018; Liang et al., 2019; Niu, Zhang, Kounios, & Liang, 2020; Peng, Gong, Beckmann, Vedaldi, & Smith, 2021; Rokicki et al., 2021; Smith et al., 2019). An example of a correction procedure is provided in Figure 1, where a correction is applied to the predictions by first fitting Y=α×Ω+β, where Y is the modelled predicted age as a function of chronological age (Ω), and *α* and *β* represent the slope and intercept. The derived values of *α* and *β* are used to correct predicted age with *Corrected Predicted Age* = *Predicted Age* + $\left[\Omega -\left(\alpha \times \Omega +\beta \right)\right]$. Delta values can then be calculated as (*Corrected delta = Corrected Predicted Age* − *Chronological Age*), which gives equivalent results to applying the correction directly to the delta values (see, e.g., Beheshti et al., 2019; de Lange & Cole, 2020; Liang et al., 2019; Smith et al., 2020), as illustrated in Figure 1.

The approach described above can be used to derive the *α* and *β* coefficients from a fit in a training set, and use them to correct the predictions or brain age deltas in an independent test set (Beheshti et al., 2019; Gong et al., 2021; Liang et al., 2019; Peng et al., 2021; Rokicki et al., 2021; Smith et al., 2019). Alternatively, the correction can be applied to the full dataset, which, although representing a scenario of data leakage, gives equivalent results to regressing out chronological age from brain age delta and using the residuals (de Lange et al., 2019; Kaufmann et al., 2019; Le et al., 2018; Richard et al., 2019; Tønnesen et al., 2020), or using age as a covariate in regressions/correlations between brain age delta and other variables of interest (Anatürk et al., 2021; de Lange, Barth, et al., 2020; Le et al., 2018). While reporting uncorrected model performance metrics and subsequently age‐correcting the delta values (or including age as a covariate in subsequent analyses) is commonly done, this yields identical statistical adjustments (de Lange & Cole, 2020) and hence does not circumvent the influence of the age‐correction on the predictions and “behind the scenes” inflation of prediction accuracy (Butler et al., 2021).

To assess the effect of age correction on performance metrics, we applied the approach described above to (i) the full UKB and Cam‐CAN models, (ii) UKB models based on subsets with different age range and sample sizes and (iii) a series of UKB and Cam‐CAN models were 0, 10, 25, 50 and 75% of the data was randomly shuffled (age values are randomly reordered across subjects), to systematically assess corrected metrics across models with different levels of initial prediction accuracy. The shuffling experiment was conducted to simulate scenarios in which the model performance shifts from more to less accurate, in order to test if the influence of the age‐bias correction on the predictions varies according to how accurate the initial model is. To test if using the coefficients from a fit in a training set to correct the predictions in an independent test set yielded different results, we split the full UKB and Cam‐CAN samples in half to produce subsets A and B. A model trained on dataset A (B) was used to make predictions in dataset B (A). A fit to predicted versus true age was performed on dataset A (B), and the coefficients *α* and *β* applied to dataset B (A) to correct the predictions. The same cross‐check was performed for the UKB models in Section 3.3.

## 3 RESULTS

### 3.1 Full models

**FIGURE 2:**
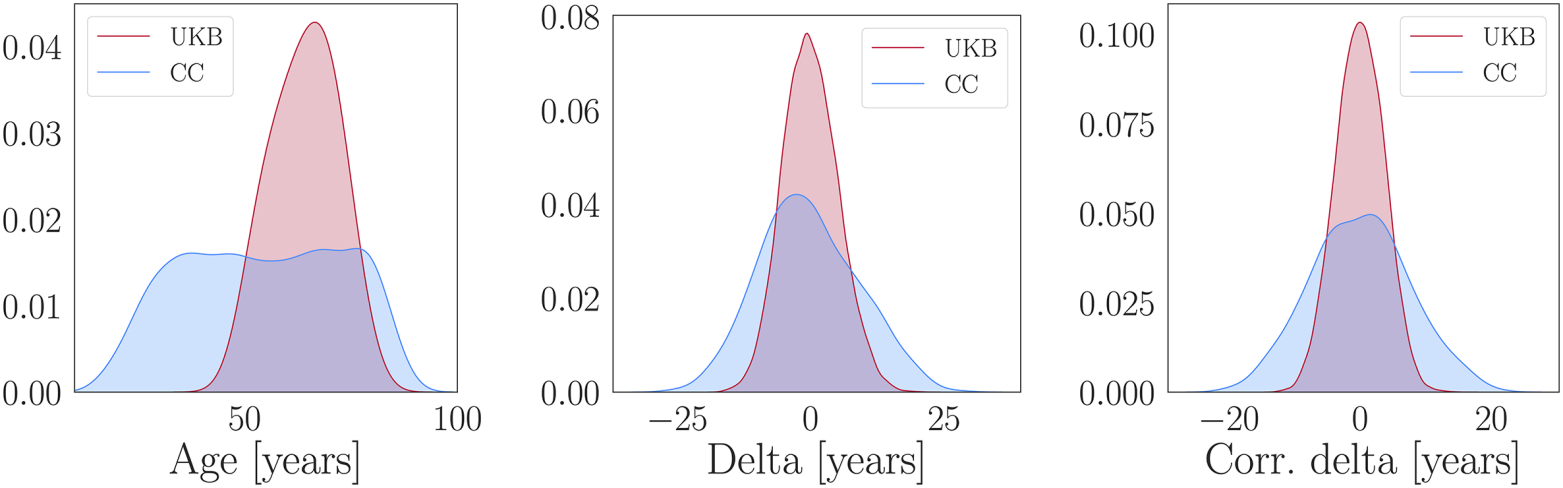
Age distributions (left plot), uncorrected brain age delta distributions (middle plot) and corrected brain age delta distributions (right plot) in UKB (red) and Cam‐CAN (CC; blue). The distributions are normalised to have the same area, and the *y*‐axes represent the density

The performance metrics for the 10‐fold cross‐validated models including the total sample size and full available age range for each dataset are provided in Table 3. Despite the smaller sample size (622 vs. 37,156 in UKB), the Cam‐CAN prediction showed larger *r* and *R* ^2^ values. The Cam‐CAN model also showed larger RMSE and MAE values due to its wider age range (18–87 vs. 45–82 in UKB). Hence, the lower RMSE/MAE values in UKB compared to Cam‐CAN are not due to better model performance, but rather reflect that predictions in samples with a narrower age range are closer to the mean age of the group, which results in lower errors/smaller brain age delta values as shown in Figure 2. All performance metrics improved for both models after age‐bias correction, as shown in Table 3. When adjusting for age‐bias using fit coefficients derived from a training set to correct the predictions in independent test sets, the results were highly comparable (Table 4). To check for potential scanning site effects (Alfaro‐Almagro et al., 2021; Solanes et al., 2021), we plotted the UKB delta distributions and calculated the correlation between predicted and true age (*r*) for each site separately. As shown in Figure S2, the results were similar across the three sites.

**TABLE 3:** The correlations (*r*) between predicted age and chronological age, *R* ^2^, root mean square error (RMSE) and mean absolute error (MAE) ± uncertainties for the age predictions including the total sample and full age range in each of the datasets

	UKB	UKB corr.	Cam‐CAN	Cam‐CAN corr.
*r*	0.728 ± 0.002	0.898 ± 0.001	0.870 ± 0.008	0.927 ± 0.005
*R* ^2^	0.529 ± 0.003	0.760 ± 0.002	0.753 ± 0.013	0.837 ± 0.011
RMSE (years)	5.169 ± 0.018	3.687 ± 0.014	9.134 ± 0.230	7.417 ± 0.186
MAE (years)	4.140 ± 0.015	2.969 ± 0.012	7.403 ± 0.217	6.001 ± 0.162

*Note*: The performance metrics are provided before and after age‐bias correction (corr).

**TABLE 4:** Performance metrics ± uncertainties provided before and after age‐bias correction (corr), where the coefficients derived from a training set are used to correct the predictions in separate test sets

	UKB	UKB corr.	Cam‐CAN	Cam‐CAN corr.
*r*	0.722 ± 0.003	0.898 ± 0.001	0.889 ± 0.008	0.930 ± 0.005
*R* ^2^	0.521 ± 0.004	0.756 ± 0.003	0.790 ± 0.014	0.844 ± 0.011
RMSE (years)	5.205 ± 0.025	3.711 ± 0.018	8.427 ± 0.234	7.260 ± 0.194
MAE (years)	4.176 ± 0.022	3.002 ± 0.015	6.797 ± 0.203	5.788 ± 0.171

*Note*: N in training and test sets = 18,578/18,578 for UKB, 311/311 for Cam‐CAN.

### 3.2 Effects of age range and sample size

This section shows model performance metrics measured in subsets with different age ranges. As a cross‐check, we repeated the age‐range tests using samples where the lower instead of upper age limit was kept constant. The results were consistent, as shown in Figures S3–S5.

#### 3.2.1 Test sets with varying age ranges; training set held constant

**FIGURE 3:**
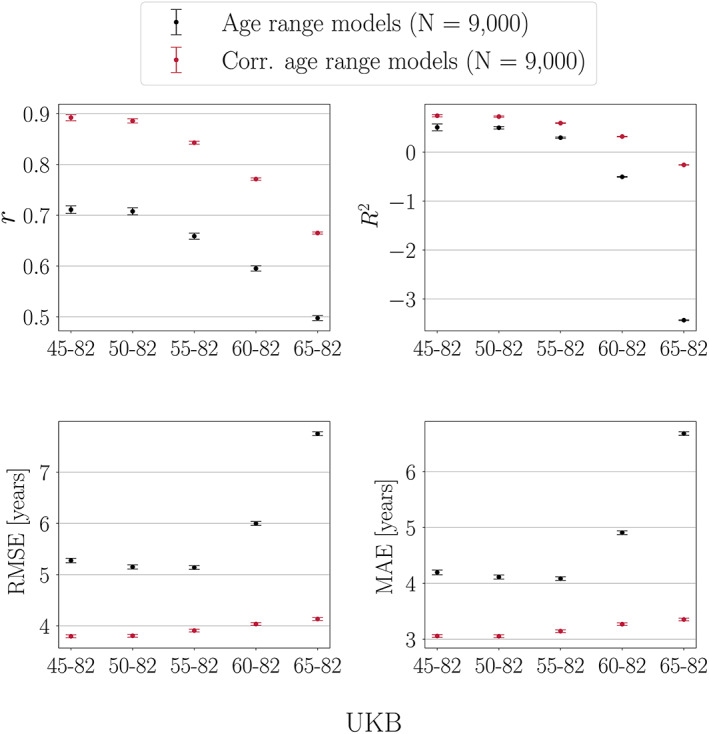
Performance metrics calculated in UK Biobank (UKB) test sets with different age ranges. Predictions are based on a model trained on the full age range. The *x*‐axes indicate the age range for each of the test sets. Sample size is kept constant across training and test sets, and represents the maximum number of participants available with the narrowest age range (65–82 years). Corr = corrected, representing the model metrics calculated post age‐bias correction

Figure 3 shows the model performance metrics calculated in UKB test sets with different age ranges when a model trained on the full age range is applied to each test set.

##### *r* and *R* ^2^ values

As seen in Figure 3, *r* values are lower when calculated in test sets with a narrower age range, even though the predictions are based on a training set including the full age‐range. The correlation coefficient is in general lower when measured in restricted ranges of a variable (Bland & Altman, 2011; Bryant & Gokhale, 1972), which is due to a smaller range in predicted and true age leading to less covariance. This also applies to the *R* ^2^ values, but *R* ^2^ is influenced by an additional effect; due to larger difference in mean age between the training and test sets, the *R* ^2^ value becomes negative for the narrowest age range. The age‐bias corrected *r* and *R* ^2^ values are generally larger for all models, and the corrected values decrease with a narrower age range. In this scenario, the prediction variance is similar across test sets, which is a result of the training set being held constant. Hence, while both corrected and uncorrected *r* and *R* ^2^ values are lower when measured in test sets with a restricted age range, low values do not imply that the brain‐predicted age estimates are invalid (prediction variance is further discussed in Section 3.3). For *R* ^2^, the test set with the narrowest age range shows the largest improvement after age‐bias correction. This is because the correction adjusts the mean age difference between the training and test sets, as further described below.

##### RMSE and MAE values

As seen in Figure 3, RMSE and MAE initially decrease as the age range is narrowed, but then show a subsequent increase in the test sets with the narrowest age range. This trend is due to two competing effects: (a) the RMSE and MAE values generally *decrease* in test sets with a narrower age range due to smaller prediction range; (b) the RMSE and MAE values *increase* with a larger mean age difference between the training and test sets. When Effect 2 becomes more prominent than Effect 1, a turning point in RMSE and MAE is observed. The mean age and delta values for the training set and each of the test sets are shown in Table 5. After age‐bias correction, the RMSE and MAE values are generally smaller for all models, with similar values across test sets as seen in Figure 3. The similar values are due to stable prediction variance across test sets (a result of the training set being held constant). As seen for *R* ^2^, the test set with the narrowest age range shows the largest improvement in RMSE/MAE after age‐bias correction, due to the adjustment of the mean difference between the training and test sets.

**TABLE 5:** Mean ± *SD* for age and model errors/brain age delta values in the training set and each of the test sets with different age ranges

	Age	Brain age delta	Corr. Brain age delta
Training set (45–82 years)	64.16 ± 7.56	0.01 ± 5.41	2.67 × 10^−14^ ± 3.80
Test set (45–82 years)	64.17 ± 7.50	−0.05 ± 5.28	8.60 × 10^−15^ ± 3.66
Test set (50–82 years)	64.48 ± 7.27	−0.32 ± 5.14	1.28 × 10^−14^ ± 3.80
Test set (55–82 years)	66.15 ± 6.12	−1.96 ± 4.75	3.19 × 10^−17^ ± 3.90
Test set (60–82 years)	68.17 ± 4.89	−4.01 ± 4.46	−1.51 × 10^−14^ ± 4.04
Test set (65–82 years)	70.64 ± 3.68	−6.42 ± 4.34	−1.89 × 10^−14^ ± 4.13

*Note*: *Corr* indicates the age‐corrected delta values. Larger mean age difference between training and test sets leads to smaller *R* ^2^ values and larger RMSE and MAE values, as shown in Figure 3.

#### 3.2.2 Training sets with varying age ranges; test set held constant

**FIGURE 4:**
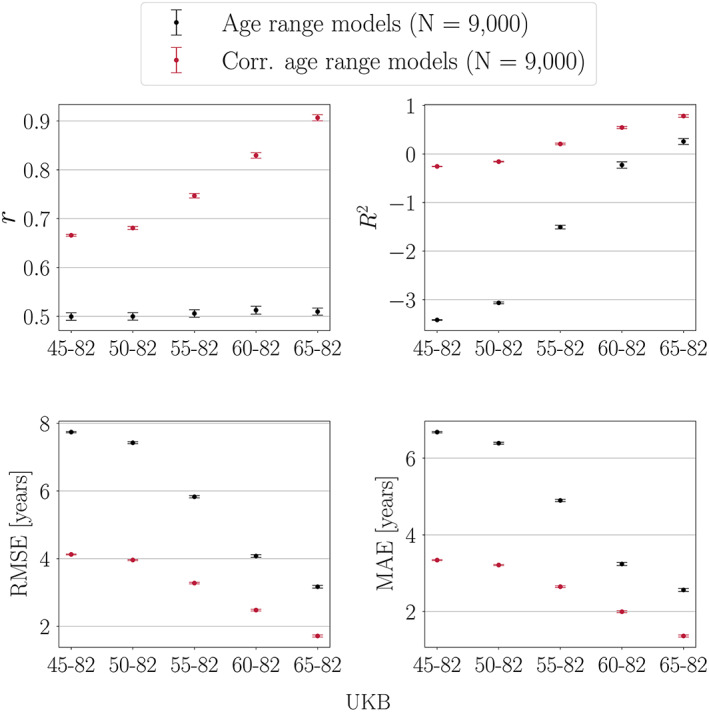
Performance metrics calculated in a UK Biobank (UKB) test set (age range = 65–82 years). Predictions are based on models trained with different age ranges. The *x*‐axes indicate the age range of the training sets applied to the same test set. Sample size is kept constant across training and test sets, and represents the maximum number of participants available with the narrowest age range (65–82 years). Corr = corrected, representing the model metrics calculated post age‐bias correction

Figure 4 shows the model performance metrics when models trained on different age ranges are applied to the same test set.

##### *r* and *R* ^2^ values

As seen in Figure 4, the uncorrected *r* values are stable for all models, although the predictions are based on training sets with different age ranges. This is because the correlation coefficient is determined by the restricted age and prediction range in the test set (which is held constant). For *R* ^2^, the uncorrected values increase substantially when the training is based on a narrower age range, due to the decreasing difference in mean age between the training and test sets (the mean age difference is largest when the training is based on the full age range, and smallest when the training is based on the narrowest age range (65–82 years) as it matches the age range of the test set (65–82 years)). After age‐bias correction, the *r* values are generally larger for all models, but the largest improvement is seen for the model where the training is based on the narrowest age range. This is due to lower prediction variance in training sets with a narrower age range: the lower the initial variance, the larger the improvement in *r* after age‐bias correction (see Section 3.3). For *R* ^2^, the largest improvement after age‐bias correction is seen for the model where the training is based on the widest age range. This is because the correction adjusts the mean age difference between training and test sets, which is largest when the training is based on the widest age range.

##### RMSE and MAE values

As shown in Figure 4, RMSE and MAE decrease when the training is based on a narrower age range. This is due to two effects: (i) lower prediction variance in models trained on a narrower age range, and (ii) decreasing mean age difference between training and test sets. After age‐bias correction, the largest improvements in RMSE and MAE are seen when the training is based on the widest age range. This is because the correction adjusts the difference in mean age between the training and test sets, which is largest when the training is based on the widest age range. Although the correction adjusts mean age differences, corrected RMSE and MAE values still decrease when training sets are based on a narrower age range. This is due to lower prediction variance with a narrower age range, which results in smaller model errors/brain age delta values (Figure 8).

#### 3.2.3 Training and test sets with equal age ranges

**FIGURE 5:**
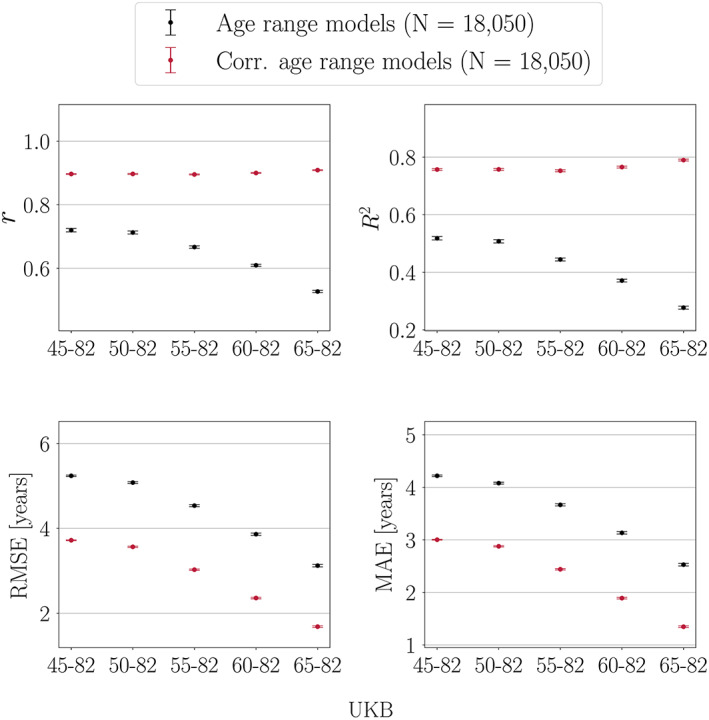
Performance metrics calculated in UK Biobank (UKB) subsets with different age ranges. Predictions are based on models trained using 10‐fold cross‐validation within each subset (age range is equal for training and test sets). The *x*‐axes indicate the age range for each of the subsets. Sample size is kept constant across subsets, and represents the maximum number of participants available with the narrowest age range (65–82 years). Corr = corrected, representing the model metrics calculated post age‐bias correction

Figure 5 shows the model performance metrics when 10‐fold cross‐validations are run within different age‐range subsets (age range is equal for training and test sets).

##### *r* and *R* ^2^ values

As seen in Figure 5, the uncorrected *r* values decrease with a narrower age range. This is due to two effects: (i) *r* is smaller in subsets with a narrower age range due to restricted age and prediction range, and (ii) the variance in predictions is smaller when the training is based on a narrower age range. Since the age range is equal for training and test sets within each subset, there are no mean age differences. Hence, *R* ^2^ values are only influenced by the same effects as *r*; variable range and variance in predictions. After age‐bias correction, the *r* values improve substantially across subsets, with the largest improvement seen for models with the lowest initial *r* values. This is due to lower prediction variance in subsets with a narrower age range (see Section 3.3). The same effect is reflected in the corrected *R* ^2^ values.

##### RMSE and MAE values

As shown in Figure 5, RMSE and MAE decrease with a narrower age range. This is due to the restricted prediction range in subsets with a narrower age range (predictions in samples with a narrower age range are closer to the mean age of the group, which equates to lower model errors/smaller brain age delta values). After age‐bias correction, the RMSE and MAE values are generally smaller for all models, but the corrected values also decrease with a narrower age range. This is due to lower variance in subsets with a narrower age range, which results in smaller model errors/delta values (Figure 8).

##### Effects of age range and sample size

**FIGURE 6:**
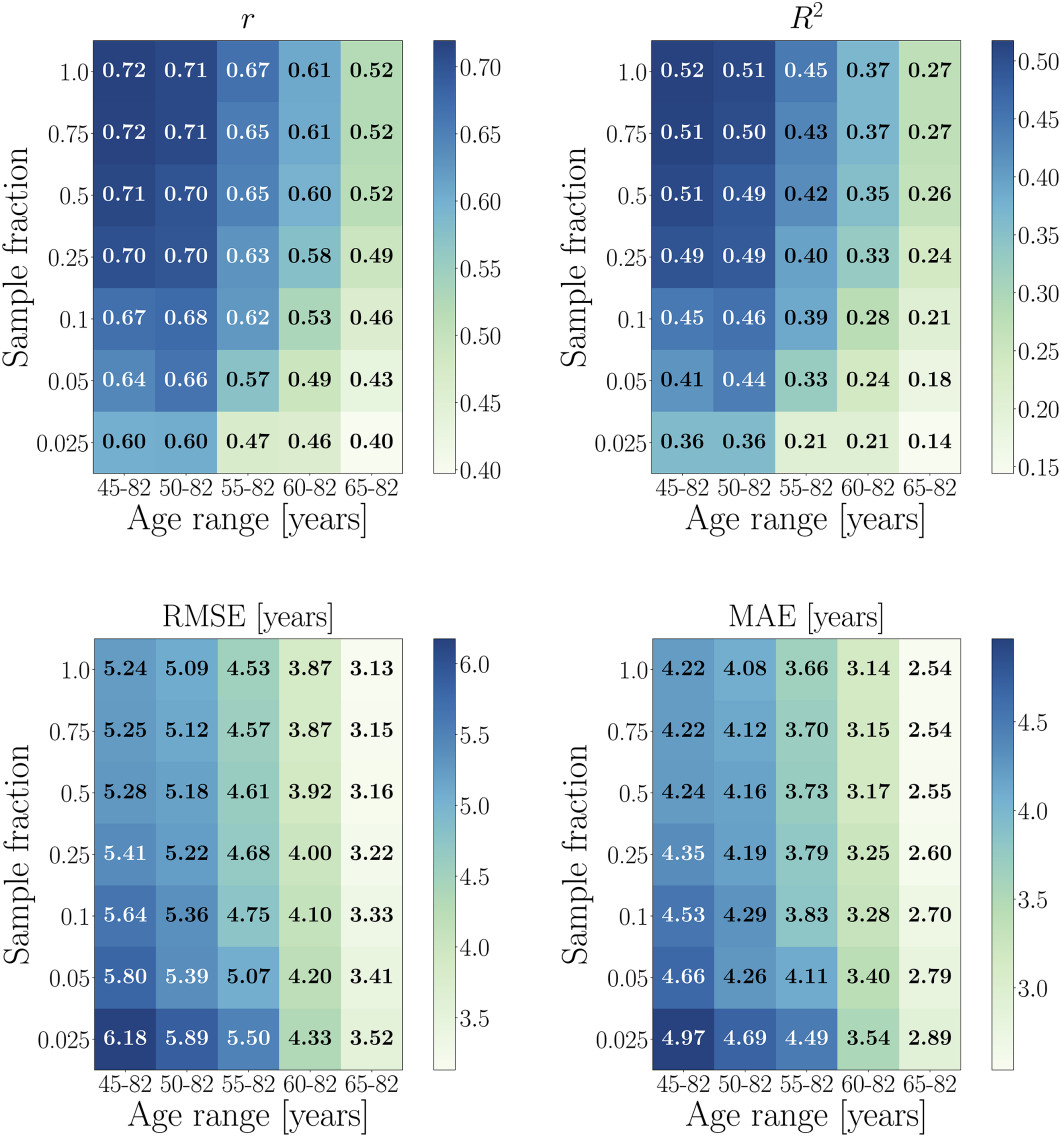
Performance metrics calculated in UK Biobank subsets with different age range and sample size. Predictions are based on 10‐fold cross‐validation models run within each age‐range subset (age range is equal for training and test sets within each subset). The *x*‐axes show the age range for each subset, while the *y*‐axes indicate the subset sizes in fractions of the maximum number of participants available with the narrowest age range; N for each sample fraction: 0.025 = 451, 0.05 = 902, 0.1 = 1,805, 0.25 = 4,512, 0.5 = 9,025, 0.75 = 13,538, 1 = 18,050

**FIGURE 7:**
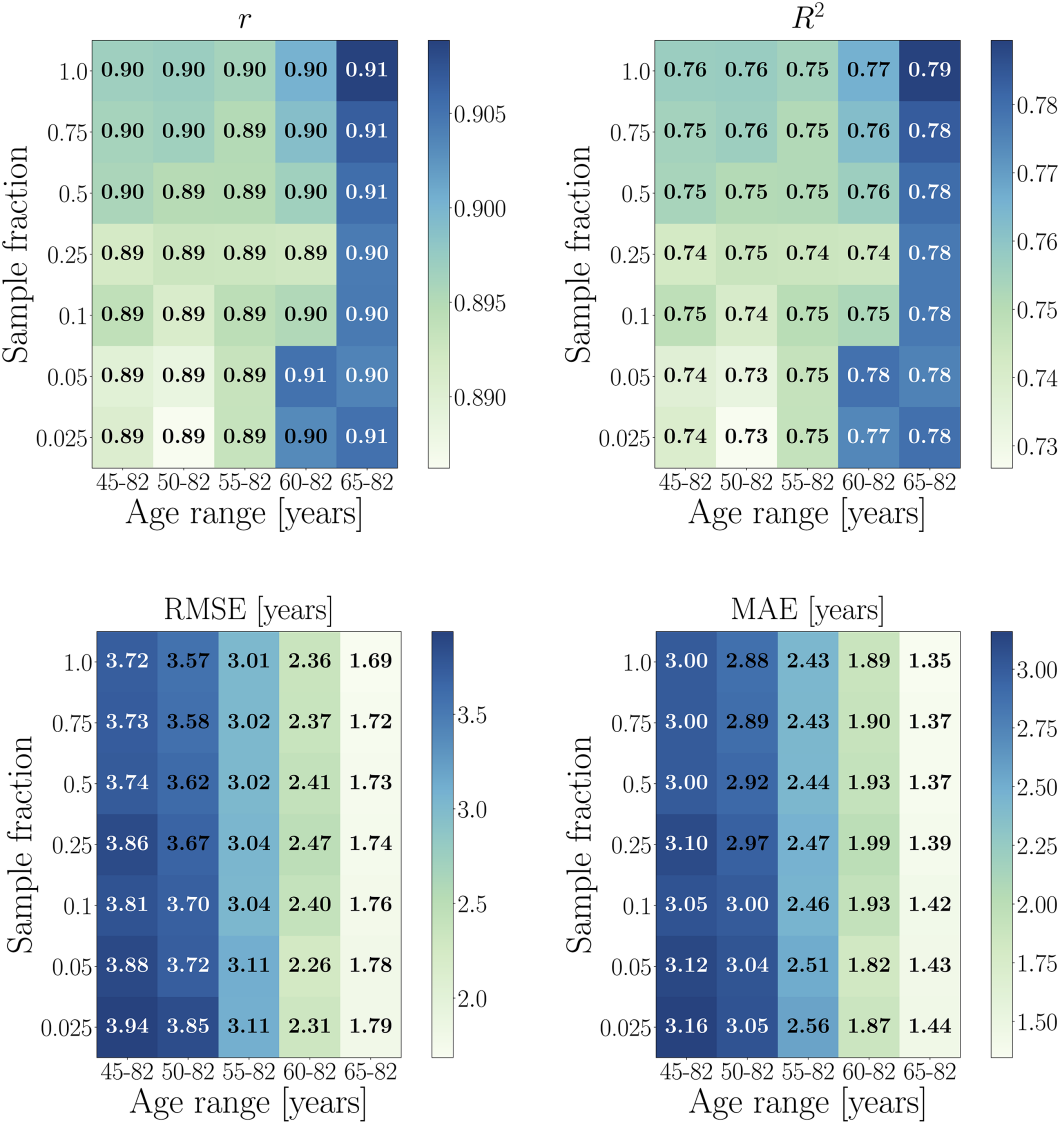
Age‐bias corrected performance metrics calculated in UK Biobank subsets with different age range and sample size. Predictions are based on 10‐fold cross‐validation models run within each age‐range subset (age range is equal for training and test sets within each subset). The *x*‐axes show the age range for each subset, while the *y*‐axes indicate the subset sizes in fractions of the maximum number of participants available with the narrowest age range (N for each sample fraction is provided in Figure 6)

As shown in Figure 6, all performance metrics improved with increasing sample size across subsets with different age ranges. Across all sample fractions, the effects of age range corresponded to the trends in Figure 5; lower uncorrected *r* and *R* ^2^ values in subsets with a narrower age range due to restricted prediction range and lower variance, and lower RMSE and MAE values in subsets with a narrower age range due to restricted prediction range. Age‐bias corrected metrics improved for all models, as shown in Figure 7.

### 3.3 Age‐bias correction applied to models with different levels of prediction accuracy

**FIGURE 8:**
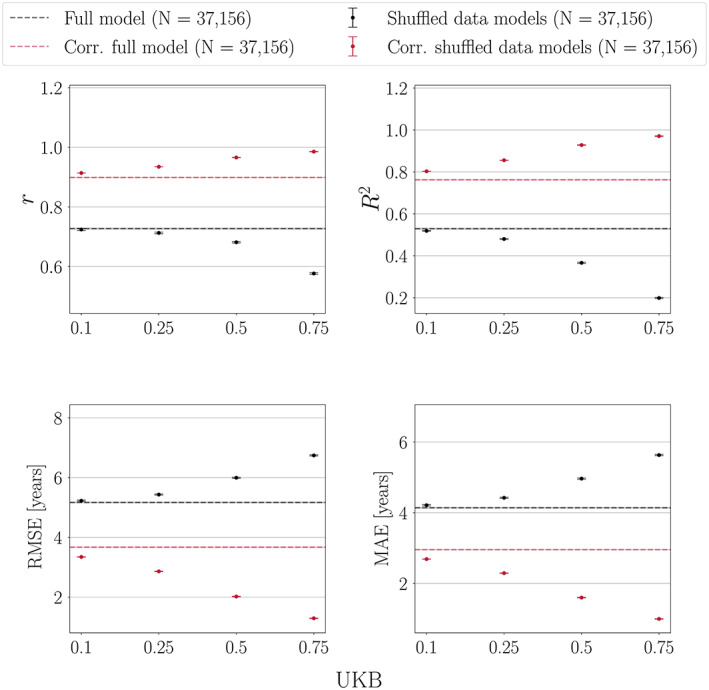
Age‐bias correction in UK Biobank (UKB) models with 0, 10, 25, 50 and 75% randomly shuffled data. All models improve after correction, and the models with the poorest initial prediction accuracy (highest fraction of shuffled data) show the largest improvement. Hence, corrected metrics may not provide a relevant representation of initial model performance

**FIGURE 9:**
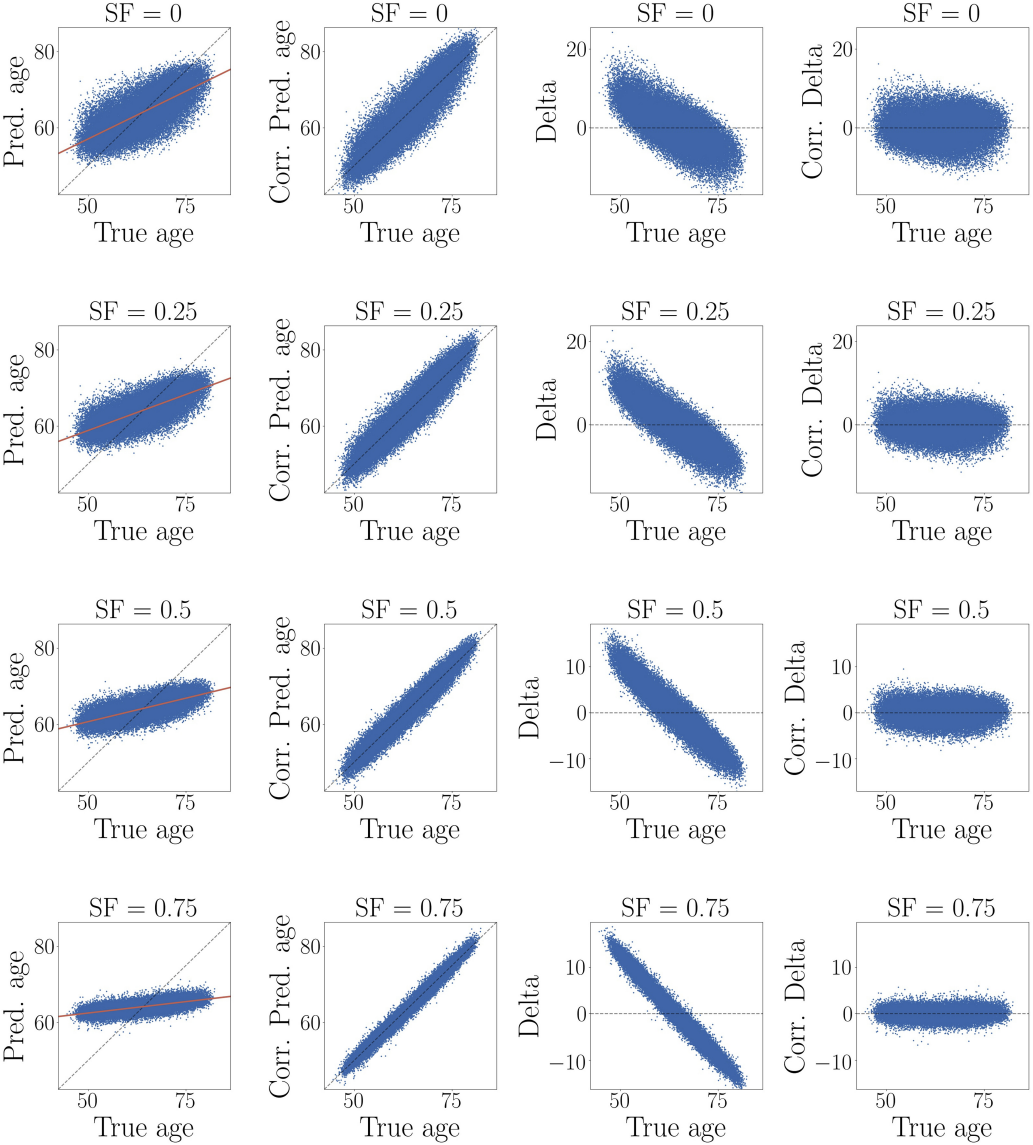
Age‐bias correction in UKB models with randomly shuffled data. SF = shuffle fraction in %. *First column*: The plots of predicted versus true age show better performance for models with lower fractions of shuffled data. The models with the best performance also display the highest prediction variance, whereas the poorly performing models show predictions that cluster around median true age, resulting in low variance. *Second column*: The relationship between predicted and true age improves after age‐bias correction, also for poorly performing models. *Third column*: Delta versus true age, illustrating the age dependence of delta. The negative slopes are due to an anti‐correlation between true age on the *x*‐axis and negative true age on the *y*‐axis, which occurs since negative true age is part of delta (*predicted age − true age*). Models with smaller slopes in predicted versus true age (first column) show larger negative slopes in delta versus true age (third column) as a result of this. *Fourth column*: Corrected delta (*corr*. *Pred age − true age*), which shows no dependence on age. Corrected delta obtained via a correction of predicted age gives equivalent results to correcting the delta values themselves for age (de Lange & Cole, 2020). Hence, while corrected delta shows no age dependence, this is due to a strong correlation between corrected predicted age and true age as a result of the correction (illustrated in Figure S17)

The results of applying the age‐bias correction to models where 0, 10, 25, 50 and 75% of the data was randomly shuffled are shown in Figure 8. All performance metrics improved after correction, and the models with the poorest initial prediction accuracy (highest fraction of randomly shuffled data) showed the largest improvement after correction due to lower variance in predictions, as shown in Figure 9. The lower variance occurs with more predictions around the median age of the sample, which is a result of the model lacking sufficient information to provide accurate predictions. For Cam‐CAN, all models improved to a similar extent after correction, as shown in Figure S6. The variance in the Cam‐CAN data was more similar across models with different shuffle fractions (Figure S7) as compared to UKB, indicating that the wider age range provides more information for the model—leading to less predictions around median age.

When using separate UKB training and test sets where the age correction parameters *α* and *β* were derived from a fit to the training set and used to correct the predictions in the test set, the results were highly comparable as shown in Figures S8 and S9. As a crosscheck, we repeated the age‐bias analysis for UKB including a quadratic age term in the correction, which showed similar results (Figure S10).

### 3.4 UKB results based on SVR instead of XGB

The UKB results based on SVR instead of XGB are shown in Section S5, Supporting Information. In line with recent studies (Dunås et al., 2021; Liang et al., 2019), we found no evidence that choice of algorithm influenced the observed patterns: the effects of age range were highly comparable (Figures S11–S13). The trends for subsets with different sample size and age range were also highly comparable, but XGB showed more stable performance across the smallest sample fractions (Figure S14). Age‐bias correction showed equivalent effects for SVR and XGB models in samples where fractions of the data were randomly shuffled (Figures S15 and S16).

## 4 DISCUSSION AND SUMMARY OF FINDINGS

Predicting age based on neuroimaging data can provide a useful marker for brain integrity and health (Cole & Franke, 2017; Cole, Marioni, et al., 2019; Kaufmann et al., 2019; Rokicki et al., 2021; Smith et al., 2020), However, the current results emphasise that the model performance metrics *r*, *R* ^2^, RMSE and MAE cannot be directly compared across different studies, as they depend on factors including age range, sample size, prediction variance and mean age differences between training and test sets.

### 4.1 Effects of age range

The results in Section 3.2 show that model performance metrics depend on cohort age range in training and test sets. Since *r* and *R* ^2^ values are lower when measured in restricted ranges of a variable (Bland & Altman, 2011; Bryant & Gokhale, 1972), these metrics can be lower when calculated in test sets with a narrow age range—also when the predictions are based on a training set with a wider age range. In this case, low *r* and *R* ^2^ values are not indicative of poor model performance or insufficient variance in brain‐predicted age estimates, but rather reflect the limited age variance in the test set. In studies where predictions are estimated in several sub‐samples, it may be useful to include the age variance of the sub‐sample with the largest age range in the calculation of performance metrics (Franke et al., 2010; Holmes, 1990), provided that the variances are similar in the sub‐sample and a matching/restricted range of the sample used. In contrast, the use of training sets with a restricted age range can potentially involve poor model performance accompanied by low prediction variance, which is further discussed in Section 4.2.

In addition to age range and prediction variance, the *R* ^2^ value is also influenced by differences in the mean age between training and test sets. Larger mean age differences lead to smaller *R* ^2^ values, as well as larger RMSE and MAE values. However, the error metrics RMSE and MAE will in general *decrease* with a narrower age range, since predictions in samples with a narrower age range are closer to the mean age of the group (which results in lower model errors/smaller brain age delta values). Hence, small model errors do not necessarily reflect better model performance, and a model based on a cohort with a wide age range may show large *R* ^2^ and *r* values accompanied by large RMSE and MAE values (as seen with Cam‐CAN versus UKB in Section 3.1). Alternative model error metrics such as Relative Squared Error (RSE), Relative Absolute Error (RAE), Median Absolute Error and weighted MAE also vary depending on age range, as shown in Section S7, Supporting Information (Figures S18–S20).

### 4.2 Age‐bias corrected versus initial model performance

The results in Section 3.3 show how statistical age‐bias corrections can inflate performance metrics by forcing an alignment between predicted and true age, leading to accurate predictions also for poorly performing models. This type of correction accounts for age‐bias and mean age differences between training and test sets, but corrected performance metrics can also conceal potential issues with low prediction variance. While reporting uncorrected model performance metrics and subsequently correcting the delta values (instead of the predictions) is common, these procedures yield equivalent corrections by shifting the estimations to the same extent since the delta value contains the prediction minus age, and age is used in the correction fit (see Figures 1 and S17; de Lange & Cole, 2020). Hence, correcting the delta values instead of the predictions does not truly circumvent inflated prediction accuracy, and corrected delta values used to assess relationships with clinical or cognitive data are not exempt from the potential variance‐related issues shown in Figures 8 and 9. Alternative correction procedures have also been applied in previous studies. For example, the method outlined in Cole et al. (2018) adjusts the slope without utilising chronological age. While this method does not inflate performance metrics, it inevitably increases the variance of the data as it divides the predicted age for each subject on the slope value (*α*) obtained from the regression fit. As an example, if we measure an intercept of 2 and a slope of 0.5, each individual's (predicted age − intercept) gets divided by 0.5. An individual with a predicted age of 50 will as a result get a corrected predicted age of 96, and an individual with a predicted age of 60 will get a corrected predicted age of 116. If the individual with a predicted age of 50 is 40 years old, the delta value goes from 10 to 56. While this is not necessarily a problem given that the scaling is usually moderate, it does complicate comparisons of mean differences in brain age, for example, between patients and controls, across studies using different correction methods (de Lange & Cole, 2020).

As recently emphasised by Butler et al. (2021), further methodological and theoretical work is critical to improve the current limitations of available age‐correction procedures. Meanwhile, inspection of uncorrected data can provide important information; for example, *r* and *R* ^2^ values calculated in test sets with a narrow age range may be low, but prediction variance may be large if the training set has a wider age range. When the age range of the training set is also restricted, low *r* and *R* ^2^ values may be due to low model performance accompanied by low prediction variance. Since age‐bias corrected predictions/delta values do not contain information about these underlying model attributes, plotting the initial fit and data points can be helpful for evaluating the validity of brain‐predicted age estimates. For example, if the relationship between the MRI input features and the dependent variable (age) is low in the training set, predictions may cluster around the median age of the sample as the model lacks sufficient information to provide accurate predictions. This would raise the question of what brain‐predicted age estimates derived from models with low prediction accuracy actually represent, and whether other types of estimates (e.g., summary scores of the imaging data that are not obtained via age prediction) may be more appropriate in the given sample.

Since structural and functional brain measures show differential variation with age across the lifespan, age prediction accuracy varies depending on input features as well as cohort characteristics. For example, we found low age prediction accuracy based on resting‐state functional MRI (fMRI) in UKB (Maglanoc et al., 2020) and the Whitehall II MRI sub‐study cohort (WHII; de Lange, Anatürk, et al., 2020). In WHII (*N* = 610, age range 60–85 years), the fMRI features showed weaker relationships with age compared to grey matter features derived from T1‐weighted scans, and this result was also replicated in a matched UKB sub‐sample in the same study. When systematically extending the UKB sub‐sample, the fMRI prediction accuracy improved with a wider age range and larger sample size, but remained consistently lower than grey‐matter based predictions in line with other UKB analyses (Cole, 2020). Such findings further emphasise the challenges of comparing model results across studies, as model performance depends on specific brain characteristics and the age span over which they are modelled. The distribution of morphometric features may also vary across different age groups due to study exclusion criteria and rates of undetected pathology among included participants. This may have an impact on prediction accuracy at different ages, as well as on inferences regarding longitudinal trajectories across the lifespan.

### 4.3 Clinical applicability

Since brain age delta values provide an estimate of deviations from expected age trajectories, this measure can be valuable for identifying differences in patients relative to healthy controls (Han et al., 2020; Kaufmann et al., 2019; Rokicki et al., 2021; Tønnesen et al., 2020). Brain‐predicted age estimates are also promising in terms of predicting prognosis in diseases such as dementia (Biondo et al., 2020; Gaser et al., 2013; Wang et al., 2019) and multiple sclerosis (Cole et al., 2020; Høgestøl et al., 2019). From a methodological point of view, prediction models can benefit from advancements such as incorporating uncertainties into the predictions (Hahn et al., 2021; Marquand et al., 2019; Peng, Gong, Beckmann, Vedaldi, & Smith, 2021). Predicted age estimates are currently represented by a single value per individual, and while MAE and RMSE values describe overall model errors, an uncertainty measure per estimate could provide a realistic accuracy range for each individual's brain‐predicted age. This could be obtained by using bootstrapping (Efron & Tibshirani, 1994): *N* (e.g., 500) different versions of the training set are created using random sampling with replacement. These training sets are used to train *N* models, which will generate a distribution of predicted age values for each subject with a mean *μ* and *SD σ*. Here, *σ* represents the uncertainty of a person's brain‐predicted age, so that it becomes possible to determine whether their chronological age falls within the confidence range *μ* ± *σ*. This could be applied to clinical contexts, where the proportion of the respective brain age delta estimates falling above a clinical risk threshold (e.g., 95 or 99%) would represent the probability for the individual to be diagnosed as at risk.

While beyond the scope of the current study, feature importance assessment can be used to identify the MRI measures that are most prominently used in the model (Salih et al., 2021; Amoroso et al., 2019; Samek, Montavon, Lapuschkin, Anders, & Müller, 2021; Vercio et al., 2020), and partial dependence plots (Friedman, 2001; Zhao & Hastie, 2021) can provide detailed information about how a specific feature contributes to the prediction (Al Zoubi et al., 2018; de Lange, Anatürk, et al., 2020). However, the most important features for age prediction in healthy controls may not necessarily overlap with the pathophysiological mechanisms of brain disorders (Bashyam et al., 2020; Rokicki et al., 2021). Hence, in clinical studies aiming to identify differences in brain tissue affected by a specific disease, modality‐specific models may provide more relevant biomarkers as compared to global models showing accurate prediction of age (Rokicki et al., 2021). Furthermore, longitudinal studies can characterise brain age trajectories over time, determining whether modifiable variables such as cardiovascular health and lifestyle behaviours serve as risk factors for the accelerated decline, and to what extent genetics and early life factors explain individual differences in brain‐predicted age (Beck et al., 2022; Elliott et al., 2019; Vidal‐Pineiro et al., 2021).

### 4.4 Conclusion

Performance metrics used for evaluating age prediction models depend on cohort and study‐specific data characteristics, and cannot be directly compared across different studies. Although some effects can be mitigated through study designs where age distributions are carefully matched across training and test sets, observed model performance in a given test set cannot be generalised to samples with different age ranges. Since age‐bias corrected metrics in general indicate high accuracy, even for poorly performing models, inspecting uncorrected results can provide important information about underlying model attributes such as prediction variance. While age prediction models have been used for more than a decade to generate imaging‐based biomarkers (Franke & Gaser, 2019), the approach continues to be developed and extended (see, e.g., Anatürk et al., 2021; de Lange, Barth, et al., 2020; Kaufmann et al., 2019; Maglanoc et al., 2020; Peng et al., 2021; Smith et al., 2020). Although not a main focus in the current study, an increasingly common scenario involves combining data from various cohorts and scanners, which poses additional challenges related to site‐ and scanner‐dependent variance (Alfaro‐Almagro et al., 2021; Solanes et al., 2021; Tønnesen et al., 2020). Improving methods for site/scanner adjustments (Bayer et al., 2021; Dinga, Schmaal, Penninx, Veltman, & Marquand, 2020), or incorporating uncertainties into the predictions (Hahn et al., 2021; Marquand et al., 2019), represent promising avenues for further developing robust and valid biomarkers for brain health and disease. As evident from the current results, clear reporting of sample characteristics and model attributes is important to enable accurate interpretation of model performance metrics in future work.

## AUTHOR CONTRIBUTIONS

Ann‐Marie G. de Lange conceptualised the study, analysed the data and wrote the first draft. All authors contributed with conceptual input and interpretations of the results, critically reviewed the manuscript drafts and approved the final manuscript.

## Supporting information


**Appendix** **S1**: Supporting Information

## Data Availability

The data are available through established access procedures for UKB (https://www.ukbiobank.ac.uk/researchers) and Cam‐CAN (https://www.cam-can.org/index.php?content=dataset). The code used for running the age prediction models is available at https://github.com/amdelange/brainage.
